# Efficacy of Auricular Acupressure in Prevention and Treatment of Chemotherapy-Induced Nausea and Vomiting in Patients with Cancer: A Systematic Review and Meta-Analysis

**DOI:** 10.1155/2021/8868720

**Published:** 2021-08-03

**Authors:** Lichan Chen, Xiaohong Wu, Xisui Chen, Chunjiao Zhou

**Affiliations:** ^1^The First Affiliated Hospital of Shantou University Medical College, Shantou, China; ^2^Shantou University Medical College, Shantou, China; ^3^The Second Affiliated Hospital of Guangzhou University of Chinese Medicine, Guangzhou, China

## Abstract

**Background:**

More than 40% of patients with cancer have reported that chemotherapy-induced nausea and vomiting (CINV) remained the most debilitating side effects of treatment even in the era of new antiemetics.

**Objective:**

The purpose of this review was to systematically evaluate the clinical effect of auricular acupressure (AA) in prevention and treatment of chemotherapy-induced nausea and vomiting.

**Methods:**

The following databases were searched: PubMed, Cochrane Library, EMBASE, the Web of Science, Chinese Biological Medicine (CBM), Chinese National Knowledge Infrastructure (CNKI), Wanfang, and VIP (from database inception to April 2020). Eligible randomized controlled trials of auricular acupressure in treating CINV were collected, including crossover randomized design study. The meta-analysis was carried out by RevMan software (5.3).

**Results:**

Totally 19 RCTs with 1449 patients met the inclusion criteria. Compared with control groups, the relief efficiency of overall CINV was enhanced by AA combined with antiemetics (RR = 1.31, CI 1.22 to 1.41, *p* ≤ 0.001). Although the therapeutic effect on acute nausea and vomiting was not obvious, AA still played an important role in reducing delayed nausea and vomiting (delayed nausea frequency: RR = 0.68, CI −1.01 to −1.35, *p* ≤ 0.001; delayed vomiting frequency: RR = 0.91, CI −1.22 to −0.61, *p* ≤ 0.001). The likelihood of adverse reactions related to antiemetics was reduced by AA combined with antiemetics (RR = 0.62, CI 0.53 to 0.74, *p* ≤ 0.001). Statistically significant association was found between AA and incidence of constipation, diarrhea, and tiredness, while there was no statistically significant association between AA and abdominal distension or headache.

**Conclusion:**

Auricular acupressure supplementation benefited delayed chemotherapy-induced nausea and vomiting as well as constipation, diarrhea, and tiredness. AA alone or AA supplementation has little effect on acute nausea and acute vomiting. There is no conclusion on whether AA alone is superior to antiemetics in the management of delayed CINV. Further studies are needed to confirm the efficacy of auricular acupressure alone in delayed CINV and anticipatory CINV. The results of this review provided the basis for further research with more rigorous study designs, adequate sample sizes, and standardized implementation to confirm the efficacy of auricular acupressure.

## 1. Introduction

Chemotherapy-induced nausea and vomiting (CINV) is one of the most debilitating side effects of cancer chemotherapy, which seriously affects patients' quality of life and treatment compliance [1]. CINV aggravates fatigue, anxiety, and depression and decreases food intake as well [2, 3]. As a consequence, chemotherapy patients who suffered CINV are failing to meet nutritional requirements [4]. Therefore, it is necessary to have good management of CINV [2–4].

Combination of antiemetics makes a valuable contribution to preventing and managing CINV in high-risk patients [5]. Without antiemetic therapy, CINV can affect 60%–80% of chemotherapy patients. Even in the era of new antiemetics, up to 40% of cancer patients develop nausea, vomiting, or both after receiving chemotherapy, and some patients have to delay or terminate chemotherapy because of severe nausea and vomiting [1,6]. CINV can be classified as acute (occurring within 24 hours of receiving chemotherapy) and delayed (occurring days 2 to 5) according to the time of occurrence [7]. The incidence of delayed CINV is higher than that of acute CINV, and the treatment is more difficult because of the rapid action and short half-life of antiemetic agent [8]. What is more, many antiemetic agents have little effect on relieving nausea [8–10]. With a deeper understanding of the pathogenesis underlying chemotherapy-induced nausea and vomiting, some antiemetic drugs with longer half-lives (e.g., second-generation 5-HT3 receptor antagonists and NK-1 receptor antagonists) have proven to be effective. However, the high cost of the drug and side effects, such as hypotension, headache, constipation, fatigue, thirst, vertigo, and diarrhea, limit repeated administration [11, 12].

Auricular acupressure (AA) is a noninvasive and well-established treatment strategy in Traditional Chinese Medicine (TCM), which could be stimulated with magnetic beads and cowherb seeds [13]. Dr. Nogier, a well-known French neurosurgeon, proposed that the auricular pits should be distributed roughly like an inverted fetus first in 1957. Therefore, the ear provides auricular acupoints corresponding to all parts of the human body, including the internal organs [14]. Stimulating auricular acupoints can improve organ function through neurohumoral pathway.

The National Comprehensive Cancer Network (NCCN) guidelines for antiemetic clinical practice and the consensus of Chinese experts both recommend acupoint stimulation as a complementary intervention to prevent CINV [7, 15]. In recent years, scholars have applied auricular acupressure to cancer chemotherapy patients to manage CINV [13, 16–33]. The drawback is that there is still a lack of multicenter, large-sample randomized controlled trials [16]. Therefore, evidence-based support is required to evaluate the efficacy of AA. Only one systematic review [34], published in 2014, explored the effect of AA on CINV. However, no meta-analysis was performed in the review; only descriptive analysis was employed. Therefore, the purpose of this review was to evaluate the efficacy of AA as a supplementary intervention for CINV by meta-analysis in order to provide evidence-based medical evidence to improve this clinical problem. This review also highlights the efficacy of vomiting and nausea separately.

## 2. Methods

This meta-analysis was reported according to the Preferred Reporting Items for Systematic Review and Meta-Analysis (PRISMA) [35].

### 2.1. Search Strategy

The following 4 English databases and 4 Chinese databases were searched: PubMed, Cochrane Library, EMBASE, the Web of Science, Chinese Biological Medicine (CBM), Chinese National Knowledge Infrastructure (CNKI), Wanfang, and VIP (from database inception to April 2020).

PICO framework was used to form clinical queries and facilitate the literature search [36] (Table 1). Relevant keywords were identified through a primary search on PubMed and Chinese Biological Medicine (CBM) to build the search strategies.

The specific search strategy was applied to the PubMed database as an example (Table 2). In addition, the snowballing process was used to increase the sensitivity of the search, whereby reference lists of included studies and previous systematic and narrative reviews.

### 2.2. Inclusion and Exclusion Criteria

RCTs (including parallel and crossover) were included, which provided an intervention of auricular acupressure with or without antiemetic medications supplementation to any age human participants undergoing chemotherapy for cancer and had used a control of either placebo or antiemetic medication. The materials used to perform the AA must be noninvasive. The primary outcomes were respective clinical efficiencies of nausea and vomiting. The secondary outcome was incidence of adverse reactions. Studies published from 2010 to 2020 in English and Chinese languages were considered for inclusion in this review.

Review, animal studies, case reports, and non-RCTs were not included. Studies were excluded in the case that the population of interest was receiving any other traditional Chinese medicine (TCM) therapies or radiation therapy. The studies which failed to offer proper outcomes to extract and the unavailable full text were also excluded.

Two reviewers (CL and WX) selected articles independently. The reference management software EndNote was used to manage all articles and remove duplicate articles. All titles and abstracts of articles were separately screened by the two reviewers to identify related studies. Then, they evaluated the full text of the related studies for inclusion. Disagreements were resolved by discussion with a third reviewer (ZC).

### 2.3. Data Extraction

Data extraction was carried out independently by two reviewers (CL and WX) using a standardized data-extraction form (Table 3). Discrepancies were resolved by a third reviewer (ZC). The form contained author, year of publication, country, study design, participants, auricular acupoints, interventions, antiemetic agent use, and measured outcomes. Missing data was obtained from the corresponding authors via e-mail if necessary.

### 2.4. Assessment of Quality and Risk Bias

The studies were selected according to inclusion and exclusion criteria. The quality and risk bias of RCTs were assessed by two reviewers (CL and WX) independently, according to the Cochrane Handbook for Systematic Reviews [37]. Discrepancies were resolved by a third reviewer (ZC). Seven domains were assessed: random allocation, allocation concealment, blind methods for subjects, blind methods for researchers, incomplete data, selective reports, and other sources of bias. In this case, other bias was defined as whether a detailed operational method had been introduced in the study due to the characteristics of auricular acupressure. Each domain was graded with “low bias risk,” “unclear,” and “high bias risk.”

### 2.5. Statistical Analysis

The RevMan 5.3 software provided by Cochrane network was used for analysis. The dichotomous data would be expressed as relative risk (RR) with 95% confidence interval (95% CI). For continuous outcomes, mean difference (MD) and standardized mean difference (SMD) with 95% CI would be calculated appropriately. Heterogeneity was assessed by a chi-square test. If *I*^2^ ≤ 50% and *p* ≥ 0.1, we considered no heterogeneity in the included study and a fixed effect model was used. If *I*^2^ > 50% and *p* < 0.1, that means heterogeneity was significant, and a random-effects model was adopted. Moreover, heterogeneity was treated using subgroup analysis or sensitivity analysis, or only descriptive analysis to investigate possible causes from clinical perspectives.

## 3. Results

### 3.1. Search Results

**Figure 1:**
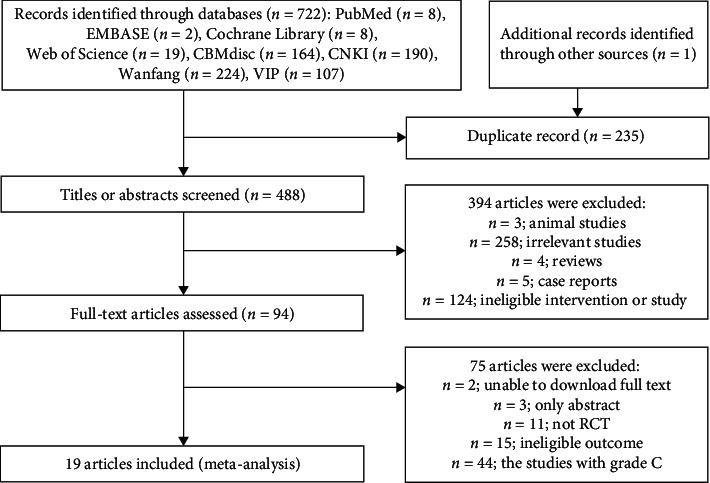
Flow diagram of study selection.

The process of search strategy and study selection is described in Figure 1. A total of 723 papers were identified through the preliminary search. Overall, 19 RCTs, containing 1449 patients, were included in the final analysis.

### 3.2. Critical Appraisal of Quality

**Figure 2:**
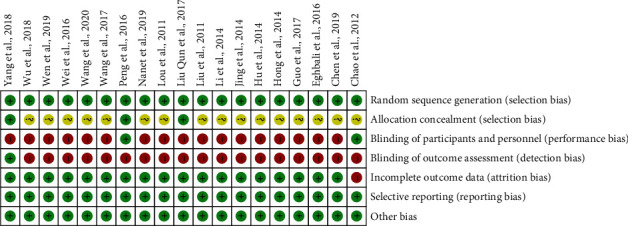
Risk of bias summary.

Figure 2 shows the risk of bias across all included studies. Randomization was mentioned in all studies. However, the majority of studies had an unclear risk of bias for allocation concealment, whereas only three studies [16–18] provided a description of concealment procedures. Due to the specificity of the interventions, blind method was difficult to achieve for participants and assessment simultaneously. Only three studies [13, 16, 18] used a single blind test. Other bias was considered as low risk because a detailed operational method of AA has been introduced in all studies.

### 3.3. Characteristics of Included Studies

Characteristics of the 19 included studies are extracted in Table 3, including 17 randomized parallel studies [16–32] and 2 randomized cross-over studies [13, 33]. Two were written in English and the other seventeen were written in Chinese. The inclusion studies were published between 2011 and 2020. The sample size was various in studies, ranging from 10 to 127. Totally 1449 patients receiving cancer chemotherapy were allocated. Only one study [13] reported the children participants. A total of 19 auricular acupoints were mentioned in the study to control CINV. The top six acupoints were stomach (100%), Shenmen (94.74%), sympathetic (73.68%), spleen (63.16%), subcortical (52.63%), and liver (31.58%). Only five studies [16, 18, 23, 25, 28] claimed the method of main acupoint plus adjunct acupoint was adopted.

### 3.4. Meta-Analysis Outcome

Different pooled data of 19 RCTs were used in meta-analysis conducted for various outcomes in cancer patients receiving chemotherapy. The efficacy of auricular acupressure on the relief efficiency and frequency and incidence of adverse reactions are shown in Table 4.

#### 3.4.1. Efficacy of Auricular Acupressure for Nausea

**Figure 3:**
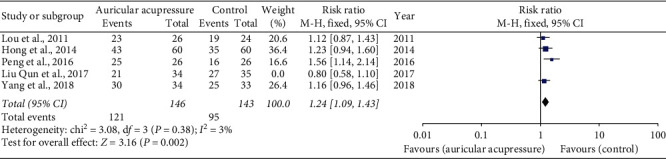
Overall nausea relief efficiency.

In terms of the relief efficiency of nausea, the only significant finding was overall nausea relief efficiency identified with sensitivity analysis. Data analysis from four of the five studies [16, 18, 20, 22] revealed that the overall nausea relief efficiency was increased by AA (RR 1.24, 95% CI: 1.09 to 1.43) (Figure 3). In sensitivity analysis, one study [17] was excluded because of significant heterogeneity with other studies. The study [17] administered intervention for auricular acupressure alone but not combined with antiemetics. Four trials [16–18, 20] evaluated the relief efficiency of acute nausea. However, there was still no statistical difference between groups after sensitivity analysis (Table 4). Three of four trials [16, 18, 20] indicated a statistically significant difference in the relief efficiency of delayed nausea between control and intervention groups. However, there was a high heterogeneity between the trials (*I*^2^ = 86%) (Table 4).

**Figure 4:**
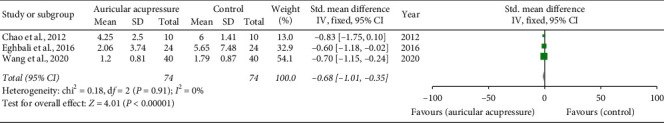
Delayed nausea frequency.

As for the nausea frequency, the significant finding was delayed nausea frequency with no statistical heterogeneity existing (*I*^2^ = 0%). Data from 3 studies [13, 32, 33] revealed that delayed nausea frequency had an evidence of significant difference (SMD −0.68, 95% CI: −1.01 to −0.35) (Figure 4). Two trials [32, 33] compared the acute nausea frequency between two groups. However, there was a heterogeneity between the trials (*I*^2^ = 63%) (Table 4).

#### 3.4.2. Efficacy of Auricular Acupressure for Vomiting

**Figure 5:**
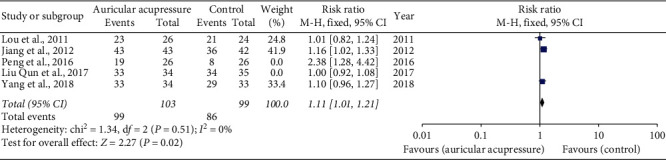
Delayed vomiting relief efficiency.

In terms of the relief efficiency of vomiting, the only significant finding was delayed vomiting relief efficiency identified with sensitivity analysis. Three of five trials [18, 20, 21] revealed that AA combined with antiemetics might more favorably reduce delayed nausea (RR 1.11, 95% CI 1.01 to 1.21) (Figure 5). Data obtained from nine studies trials [16–18, 20–22, 27, 28, 30] indicated a statistically significant difference in overall vomiting relief efficiency between two groups (RR 1.14, 95% CI 1.02 to 1.28). However, there was a heterogeneity between the trials (*I*^2^ = 56%). Five trials compared the acute vomiting relief efficiency between the two groups, whereas there was still no statistical difference after sensitivity analysis (Table 4).

**Figure 6:**
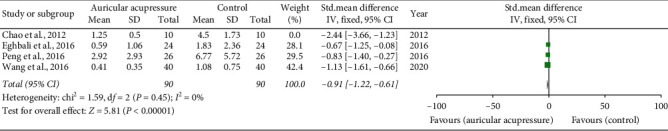
Delayed vomiting frequency.

As for the vomiting frequency, there was a significant difference in the delayed vomiting frequency with sensitivity analysis. Delayed vomiting frequency was reduced by AA, based on data analysis from three of four trials [16, 32,33] (*I*^2^ = 0%, SMD −0.91, 95% CI −1.22 to −0.61) (Figure 6). Sensitivity analysis: one trial [13] in which participants were children was excluded. The meta-analysis showed there was no statistically significant difference in overall vomiting frequency and the acute vomiting frequency between the two groups (Table 4).

#### 3.4.3. Overall Efficiency of CINV

**Figure 7:**
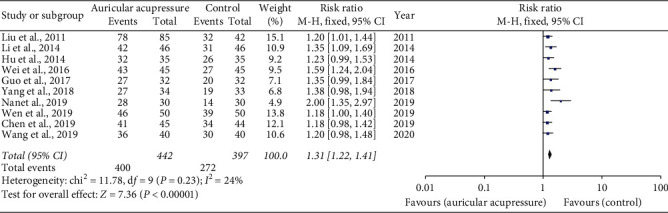
Overall efficiency of CINV.

Ten trials [18, 19, 23–26, 29–32] (*n* = 839) evaluated the overall efficiency of CINV. Given *I*^2^ < 50%, a fixed effect model was employed. There was a significant difference in the overall efficiency of CINV (RR 1.31, 95% CI 1.22 to 1.41) (Figure 7). This result indicated that chemotherapy-induced nausea and vomiting could be improved by auricular acupressure.

#### 3.4.4. Incidence of Adverse Reactions

**Figure 8:**
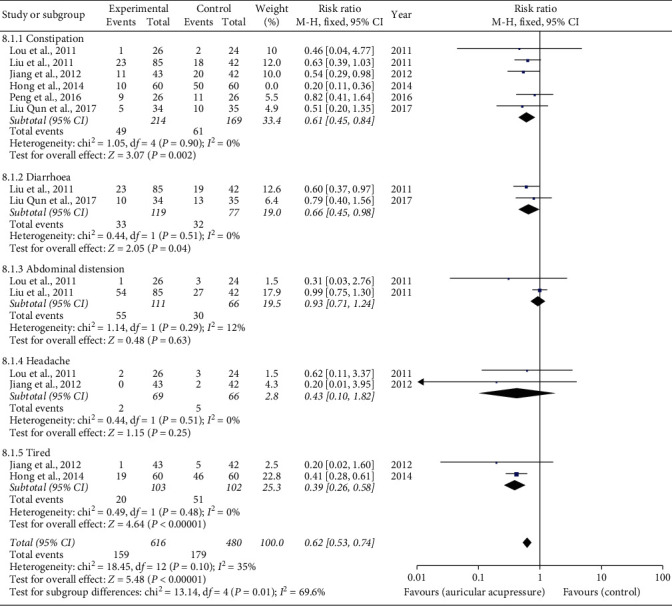
Incidence of adverse reactions.

Six trials [16, 17, 19–22] (*n* = 503) recorded the incidence of constipation. There was a statistically significant difference in the incidence of constipation with sensitivity analysis (RR 0.61, 95% CI 0.45 to 0.84) (Figure 8).

Two studies [17, 19] were pooled for the results of diarrhoea. No statistical heterogeneity existed. There was a significant difference in the incidence of diarrhoea between the auricular acupressure group and the control group (RR 0.66, 95% CI 0.45 to 0.84) (Figure 8).

The incidence of abdominal distension was compared in two studies [19, 20]. And two studies [17, 19] reported the results of headache. However, data from these studies both found no significant difference between control and intervention groups with a low statistically significant heterogeneity (Figure 8).

Two studies reported the incidence of tired [19,20], with a statistically significant difference between intervention and control groups (Figure 8).

## 4. Discussion

Although antiemetics have been widely proved to be effective on CINV, the unavoidable side effects remain a challenge to medical staff [1]. There are also reports that the incidence of delayed CINV is higher than acute CINV due to short half-life of antiemetic agent [8]. Moreover, many antiemetic agents make little contribution to relieving nausea [8–10]. To deal with these problems, behavioral therapies and acupuncture have been applied as supplementation therapies [38, 39]. Auricular acupressure was recognized as a promising therapy for its noninvasion and low cost. Though more and more original studies [13, 16–33] indicated the effectiveness of AA in controlling the CINV in patients with cancer, there is still a lack of powerful evidence because of the small sample and limitations of the outcomes in each study. No meta-analysis was performed in the previous review. The purpose of this meta-analysis was to evaluate the efficacy of AA as a supplementary intervention for CINV and find the efficacy for nausea and vomiting separately.

This systematic review included 17 randomized parallel studies [16–32] and 2 randomized cross-over studies [13, 33], involving a total of 1449 patients. It made a conclusion that compared with antiemetics alone, AA supplementation combined with standard antiemetics was more effective in preventing and treating CINV. It may be related to the stimulation of auricular acupoints which affect the neurohumoral pathway associated with CINV. These findings were consistent with conclusions made in the previous systematic reviews [34] on the topic. AA supplementation was suggested to have credible beneficial effects on reducing delayed nausea and vomiting, as well as significant improvements on constipation, diarrhea, and tiredness which were related to antiemetics. However, it remained inconclusive as to whether AA benefits acute nausea, acute vomiting, abdominal distension, and headache.

Nausea, unlike vomiting, is a graded response with a dynamic threshold influenced by a variety of factors, which makes it more difficult to prevent than vomiting. However, it was often ignored by clinicians and researchers compared to vomiting. This review found that AA changed patterns of nausea occurrence and duration. Four studies [16, 18, 20, 22] revealed that the overall nausea relief efficiency was increased by AA combined with antiemetics (RR 1.24, 95% CI: 1.09 to 1.43), while two studies [17, 22] administered intervention for auricular acupressure alone but not combined with antiemetics. In spite of no significant differences being stated between the AA group and antiemetics group, one of the studies [22] stated that AA had a better effect on nausea than ondansetron. These results supported the hypothesis that taking AA as a supplementation further improved the level of control of nausea. One more significant finding was that delayed nausea frequency was reduced by 32% with no statistical heterogeneity existing (*I*^2^ = 0%) from 3 studies [13, 32, 33]. Kenward et al. [40] reported that nausea was a subjective sensation incorporating emotional and affective components in addition to the physiological response. Continuous stimulation of the AA might tranquilize the mind, sooth the nerves, and facilitate serenity. In addition, since nausea usually precedes vomiting, better management of nausea might prevent vomiting.

As for vomiting, it is an all-or-nothing event occurring when stimuli surpass the threshold required to activate the vomiting reflex [40]. That prompted when the neuronal signals were reduced to below the threshold required, vomiting could be prevented. Five comparative studies [16–18, 20, 21] evaluated the therapeutic effect of AA for either acute or delayed vomiting. There were no significantly better outcomes for acute vomiting in the intervention groups compared to the control groups. Three of five studies [18, 20, 21] revealed that AA combined with antiemetics might more favorably reduce delayed nausea (RR 1.11, 95% CI 1.01 to 1.21). One study [17] which reported the comparisons of AA alone versus antiemetics alone was excluded to reduce heterogeneity. However, the study [17] showed antiemetics was more effective than AA alone in acute vomiting while it had no advantages in delayed vomiting. It further revealed the curative effect of antiemetics and the lasting effect of AA. Another study [16] which was excluded to reduce the heterogeneity had administered intervention for positive reacted point in AA combined with antiemetics. The study showed the efficiency of the intervention group was more than twice that in the control group (RR 2.38, 95% CI 1.28 to 4.42). This finding suggested that further well-designed studies with intervention for positive reacted points were available to improve the efficacy.

Six studies [16, 17, 19–22] explored the incidence of adverse reactions related to antiemetics. Total incidence of adverse reactions in the intervention group was lower than the control group [25.8% (159/616) vs. 37.3% (179/480)]. The results of meta-analysis showed that the incidence of constipation, diarrhea, and tiredness was decreased with auricular acupuncture intervention. That may be due to the therapeutic effects of AA on the adverse reactions related to antiemetics. Another reason has been proved in four studies [16–20] was that the use of AA reduced the doses of antiemetics. There were no significant differences in abdominal distension and headache between the two groups. That may be due to the selection of different antiemetics and the difference of chemotherapy regimens. What is more, the selection of acupoints was also related to efficacy.

In this review, a total of 19 auricular acupoints were used to control CINV. As for acupoints selection, no uniform standards were published. Five studies [16, 18, 23, 25, 28] recommended the scheme of main acupoint plus adjunct acupoint. Stomach, Shenmen, sympathetic, spleen, subcortex, and liver were the most popular selected acupoints. Stomach, located on where the crus helicis disappears, is a main acupoint for treating gastrointestinal disorders. Shenmen, which could be found in the apex of the triangular fossa, is the second acupoint commonly referred to controlling nausea and vomiting by tranquilizing the mind, facilitating serenity, and soothing the nerves. Sympathetic can regulate the responsiveness of the sympathetic and parasympathetic nervous system and relieve CINV caused by vagus nerve peripheral stimulation. Spleen is connected with the stomach internally and externally. Subcortex can adjust gastrointestinal function, playing a role in antivomiting and sedation by stimulating the cerebral cortex. Liver is mainly responsible for promoting the free flow of Qi [27]. From the above, the principles of AA can be explained in two main lines of research. Firstly, based on TCM, the stimulation of AA regulates Qi and blood in the body, activates meridians and collaterals, and finally achieves the balance of yin-yang and maintains the function of internal organs [41]. Secondly, according to neuroembryonic theory, Dr. Nogier viewed the ear as a simulation of an inverted fetus within the womb, which provides acupressure points corresponding to different parts of the human body [42]. The levels of cortisol, 5-hydroxytryptamine, and norepinephrine could be modulated by AA in neural pathways [43].

## 5. Limitations

This review has the following limitations. The first concerns the inevitably clinical heterogeneity between the included studies, which decreases confidence in the result. Different chemotherapy and antiemetic regimens, as well as cancer types and participant samples, may be the sources for clinical heterogeneity. Secondly, the lack of standardized implementation of AA in chemotherapy patients resulted in different clinical outcomes, specifically, the principle acupoints selection, duration of pressing, frequency of pressing, frequency of ear paste replacement, and qualification of the implementer. That suggests that standardized implementations will need to be built in the future. Thirdly, only one study assessed auricular acupressure to prevent/treat CINV in children with cancer. And the evidence is not powerful enough because of the small sample sizes. More multicenter, large sample, high-quality randomized controlled studies should be conducted in children. Moreover, publication bias was not able to be assessed and the review was also limited by the small number of available studies that reported the outcomes of interest.

## 6. Conclusions

In conclusion, auricular acupressure supplementation benefited delayed chemotherapy-induced nausea and vomiting as well as constipation, diarrhea, and tiredness. AA alone or AA supplementation has little effect on acute nausea and acute vomiting. Based on the existing studies, we recommend AA as a complementary therapy for the management of CINV, especially for patients with breakthrough CINV, which also reduces some adverse reactions of antiemetics. There is no conclusion on whether AA alone is superior to antiemetics in the management of delayed CINV. Further studies are needed to confirm the efficacy of auricular acupressure alone in delayed CINV and anticipatory CINV, which can greatly interfere with a patient's quality of life and hamper their willingness to continue treatment. The results of this review provided the basis for further research with more rigorous study designs, adequate sample sizes, and standardized implementation to confirm efficacy of auricular acupressure.

## Figures and Tables

**Table 1:** PICO framework.

Population	Patients receiving cancer chemotherapy
Intervention	Auricular acupressure, auricular acupressure combined with antiemetics
Comparison	Antiemetics
Outcome	Nausea relief efficiency, nausea frequency, vomiting relief efficiency, vomiting frequency, overall efficiency of CINV, and incidence of adverse reactions

**Table 2:** Example of PubMed search strategy.

ID	Search strategy	Result
#1	Search: (“acupuncture, ear”[mesh]) OR ((((((((((((acupunctures, ear[title/abstract]) OR (auricular acupuncture[title/abstract])) OR (ear acupuncture[title/abstract])) OR (acupuncture, auricular[title/abstract])) OR (auricular acupressure[title/abstract])) OR (auricular pressing[title/abstract])) OR (auricular plaster[title/abstract])) OR (auricul*∗*[title/abstract])) OR (auricular acupoint*∗*[title/abstract])) OR (auricular point*∗*[title/abstract])) OR (auricular therapy[title/abstract])) OR (ear buried seeds[title/abstract]))	1,270
#2	Search: (“drug therapy”[mesh]) OR ((((((((therapy, drug[title/abstract]) OR (drug therapies[title/abstract])) OR (therapies, drug[title/abstract])) OR (chemotherap*∗*[title/abstract])) OR (pharmacotherap*∗*[title/abstract])) OR (cancer*∗*[title/abstract])) OR (antineoplastic agents[title/abstract])) OR (drug therap*∗*[title/abstract]))	3,349,312
#3	Search (((“vomiting”[mesh]) OR emesis[title/abstract]) OR “nausea”[mesh]))	42,016
#4	#1 AND #2 AND #3	8

**Table 3:** Basic characteristics of inclusion literature.

Reference and year	Country	Study design	Participants	Tumor category	Auricular acupoints	Materials	Interventions	Antiemetics	Outcomes
Lou et al., 2011 [20]	China	Parallel RCT	50	Various cancers	Shenmen, stomach, subcortex, spleen, sympathesis, ear center	Cowherb seeds	AA + antiemetics	Granisetron	(i); (iii); (vi)
Liu et al., 2011 [19]	China	Parallel RCT	127	Lung cancer	Lung, stomach, Shenmen, spleen, diazoma, subcortex, large intestine	Cowherb seeds	AA + antiemetics	Granisetron/ondansetron	(v); (Vi)
Chao et al., 2012 [13]	Taiwan, China	Crossover placebo RCT	10	Na	Shenmen, stomach, cardia, sympathesis, subcortex,	Seeds	AA + antiemetics	Granisetron/ondansetron	(ii); (iv)
Jiang et al., 2012 [21]	China	Parallel RCT	85	Na	Stomach, Shenmen, spleen, sympathesis, large intestine	Cowherb seeds	AA + antiemetics	Granisetron	(iii); (vi)
Hong et al., 2014 [22]	China	Parallel RCT	120	Breast cancer	Stomach, Shenmen, spleen, sympathesis, subcortex	Cowherb seeds	AA	Ondansetron	(i); (iii); (vi)
Li et al., 2014 [24]	China	Parallel RCT	91	Lung cancer	Stomach, Shenmen, sympathesis	Cowherb seeds	AA + antiemetics	Ondansetron + DXM	(v)
Hu et al. 2014 [23]	China	Parallel RCT	70	Various cancers	M: stomach, Shenmen, sympathesis; A: spleen, liver	Cowherb seeds	AA + antiemetics	Tropisetron	(v)
Eghbali et al., 2016 [33]	Iran	Crossover RCT	96	Breast cancer	Point zero, stomach, brainstem, Shenmen, cardia	Seeds	AA + standard medications	Standard medications	(ii); (iv)
Peng et al., 2016 [16]	China	Parallel RCT	52	Cervical/endometrial/ovarian cancer	M: Shenmen, diazoma, stomach, cardia, occiput, subcortex, esophagus; A: sympathesis, spleen, liver	Cowherb seeds	AA + antiemetics	Ondansetron + DXM	(i); (iii); (iv); (vi)
Wei et al., 2016 [25]	China	Parallel RCT	90	Na	M: stomach, Shenmen, spleen; A: liver	Cowherb seeds	AA + antiemetics	Azasetron	(v)
Guo et al., 2017 [26]	China	Parallel RCT	64	Gastric cancer	Shenmen, sympathesis, stomach, large intestine, small intestine, Ashi point	Cowherb seeds	AA + antiemetics	Granisetron	(v)
Liu, 2017 [17]	China	Parallel RCT	70	Cervical/endometrial/ovarian cancer	Stomach, liver, small intestine, Shenmen, endocrine	Cowherb seeds	AA	Ondansetron	(i); (iii); (iv); (vi)
Wang et al., 2017 [27]	China	Parallel RCT	52	Bone cancer	Stomach, subcortex, ear center, Shenmen, spleen, sympathesis	Cowherb seeds	AA + antiemetics	Ondansetron	(ii); (iii); (iv)
Wu et al., 2018 [28]	China	Parallel RCT	86	Lung cancer	M: stomach, cardia, subcortex A: liver, sympathesis	Cowherb seeds	AA + antiemetics	Ondansetron	(Iii)
Yang, 2018 [18]	China	Parallel RCT	67	Breast cancer	M: stomach, Shenmen, subcortex, sympathesis; A: liver, spleen	Cowherb seeds	AA + antiemetics	Ondansetron	(i) (iii) (v)
Chen et al., 2019 [29]	China	Parallel RCT	89	Colorectal cancer	Sympathesis, Shenmen, spleen, stomach	Cowherb seeds	AA + antiemetics	Azasetron + DXM	(v)
Nan et al., 2019 [30]	China	Parallel RCT	60	Bone cancer	Stomach, ear center, sympathesis, spleen, Shenmen, subcortex	Cowherb seeds	AA + antiemetics	Ondansetron	(iii); (v)
Wen et al., 2019 [31]	China	Parallel RCT	100	Various cancers	Shenmen, stomach, spleen	Cowherb seeds	AA + antiemetics	Azasetron	(v)
Wang et al., 2020 [32]	China	Parallel RCT	80	Cervical cancer	Shenmen, sympathesis, stomach, subcortex, spleen	Cowherb seeds	AA + antiemetics	Ondansetron	(v)

RCT: randomized controlled trial; Na: not available; M: main points; A: adjunct points; AA: auricular Acupressure; DXM: dexamethasone. Outcomes: (i) nausea relief efficiency; (ii) nausea frequency; (iii) vomiting relief efficiency; (iv) vomiting frequency; (v) overall efficiency of CINV; and (vi) incidence of adverse reactions.

**Table 4:** Efficacy of auricular acupressure for nausea and vomiting outcomes.

Outcome	Inclusion of studies	Sample size	Heterogeneity	Analysis model	RR^a^/SMD^b^ (95% CI)	*p* value
*Nausea relief efficiency*						
Overall nausea relief efficiency	5	358	*I*^2^ = 3%; *p* = 0.38^c^	Fixed	1.24^a^ (1.09 to 1.43)	**0.002** ^**d**^
Acute nausea relief efficiency	4	238	*I*^2^ = 0%; *p* = 0.40^c^	Fixed	1.08^a^ (0.95 to 1.22)	0.23
Delayed nausea relief efficiency	4	238	*I*^2^ = 86%; *p* ≤ 0.001	Random	1.30^a^ (0.92 to 1.84)	0.14

*Nausea frequency*						
Acute nausea frequency	2	128	*I*^2^ = 63%; *p* = 0.10	Random	−0.18^**b**^ (−0.77 to 0.41)	0.55
Delayed nausea frequency	3	148	*I*^2^ = 0%; *p* = 0.91	Fixed	−0.68^**b**^ (−1.01 to −0.35)	<**0.001**

*Vomiting relief efficiency*						
Overall vomiting relief efficiency	9	634	*I*^2^ = 56%; *p* = 0.02	Random	1.14^a^ (1.02 to 1.28)	**0.02**
Acute vomiting relief efficiency	5	323	*I*^2^ = 0%; *p* = 0.64^c^	Fixed	1.05^a^ (0.93 to 1.18)	0.44
Delayed vomiting relief efficiency	5	323	*I*^2^ = 0%; *p* = 0.51^c^	Fixed	1.11^a^ (1.01 to 1.21)	**0.02**

*Vomiting frequency*						
Overall vomiting frequency	2	89	*I*^2^ = 91%; *p* ≤ 0.001	Random	−0.67^b^ (−2.61 to 1.27)	0.50
Acute vomiting frequency	3	180	*I*^2^ = 0%; *p* = 0.40	Fixed	−0.18^b^ (−0.47 to 0.12)	0.23
Delayed vomiting frequency	4	200	*I*^2^ = 0%; *p* = 0.45^c^	Fixed	−0.91^b^ (−1.22 to −0.61)	<**0.001**
Overall efficiency of CINV	10	839	*I*^2^ = 24%; *p* = 0.23	Fixed	1.31^a^ (1.22 to 1.41)	<**0.001**

*Adverse reactions*						
Overall incidence	6	503	*I*^2^ = 35%; *p* = 0.10	Fixed	0.62 (0.53 to 0.74)	<**0.001**
Constipation	6	503	*I*^2^ = 0%; *p* = 0.90^c^	Fixed	0.61 (0.45 to 0.84)	**0.002**
Diarrhoea	2	196	*I*^2^ = 0%; *p* = 0.51	Fixed	0.66 (0.45 to 0.98)	**0.04**
Abdominal distension	2	177	I^2^ = 12%; *p* = 29	Fixed	0.93 (0.71 to 1.24)	0.63
Headache	2	135	I^2^ = 0%; *p* = 0.51	Fixed	0.43 (0.10 to 1.82)	0.25
Tiredness	2	205	*I*^2^ = 0%; *p* = 0.48	Fixed	0.39 (0.26 to 0.58)	<**0.001**

^a^RR: risk ratio; ^b^MD: mean difference; ^c^improved with sensitivity analysis; and ^d^statistically significant results are highlighted in bold.
